# Extrusion of the medial meniscus is a potential predisposing factor for post-arthroscopy osteonecrosis of the knee

**DOI:** 10.1186/s12891-021-04730-7

**Published:** 2021-10-05

**Authors:** Jun Yamaguchi, Kazunori Yasuda, Eiji Kondo, Takuma Kaibara, Daisuke Ueda, Koji Yabuuchi, Jun Onodera, Norimasa Iwasaki, Mitsuru Saito, Tomonori Yagi

**Affiliations:** 1 Sports Medicine and Arthroscopy Center, Yagi Orthopaedic Hospital, 3-Jo 5-Chome, Nishino, Nishi-ku, Sapporo, 063-0033 Japan; 2 Department of Orthopaedic Surgery, Hokkaido University Graduate School of Medicine, Sapporo, Japan; 3 Department of Orthopaedic Surgery, The Jikei University School of Medicine, Tokyo, Japan; 4 Center for Sports Medicine, Hokkaido University Hospital, Sapporo, Japan

**Keywords:** Osteonecrosis of the knee, Arthroscopic meniscectomy, Meniscal extrusion, Meniscal location, Medial meniscus, Posterior root tear, Radial tear

## Abstract

**Background:**

Post-arthroscopic osteonecrosis of the knee (PAONK) is a rare condition. No studies have analyzed the relationship between the meniscus extrusion and PAONK. The purpose of this retrospective study is to test a hypothesis that the degree of the medial meniscus (MM) extrusion might be significantly greater in the knees with PAONK than in the matched control knees both before and after the meniscectomy.

**Methods:**

Ten knees with PAONK were detected out of a total of 876 knees which had undergone arthroscopic partial meniscectomy of the MM. Ten matched control knees were randomly selected out of the remaining 866 knees without PAONK. The clinical data of these 20 patients were retrospectively collected from the medical records. To evaluate the location of the menisci on the joint line, Extrusion width and Inner width were defined on a coronal section of magnetic resonance imaging (MRI). The intra- and inter-rater reliability was evaluated by calculating the intra- and inter-class coefficients. Statistical comparisons between the 2 groups were made using the 3 non-parametric tests.

**Results:**

Before the meniscectomy, the Extrusion width of the MM (mean 4.7 ± 1.4 mm) was significantly greater than that (3.0 ± 1.3 mm) in the Control group (*P* = 0.0195). In the MRI taken in a range from 3 to 50 weeks after the meniscectomy, the Extrusion width of the MM (5.9 ± 1.1 mm) in the PAONK group was significantly greater than that (3.4 ± 1.4 mm) in the Control group (*P* = 0.0009), and the Inner width of the MM (0.6 ± 1.7 mm) in the PAONK group was significantly less than that (3.9 ± 1.0 mm) in the Control group (*P* = 0.0001).

**Conclusion:**

A significant relationship was found between the degree of the MM extrusion and the onset of PAONK. This study suggested that the extrusion of the MM is a potential predisposing factor for PAONK.

## Background

Osteonecrosis of the knee was first reported by Ahlbäck et al. [1] in 1968. Currently, it has been classified into 3 different categories, spontaneous osteonecrosis (SPONK), secondary osteonecrosis, and post-arthroscopic osteonecrosis of the knee (PAONK) [2]. Concerning PAONK, Brahme et al. [3] described the first report entitled “osteonecrosis of the knee after arthroscopic surgery” in 1991. Later, similar pathological conditions were reported as “postmeniscectomy osteonecrosis” [4–6]. In 2007, Pape et al. [7] reviewed 47 previously reported cases of these pathological conditions, and they named them “PAONK”. Thus, PAONK is defined as a type of knee osteonecrosis that occurs in patients who underwent arthroscopic knee surgery. Clinical diagnosis of PAONK is confirmed with magnetic resonance imaging (MRI) [8–10]. The major requirement in diagnosing PAONK is to confirm that any evidence of any evidence of bone marrow edema (BME) or osteonecrosis was absent in the MRI taken immediately before the meniscectomy [2].

PAONK is a rare condition so that the incidence has been reported to be 0.2–1.5% of the knees which underwent arthroscopic surgery [5, 6]. Therefore, the etiology of PAONK remains unclear. Namely, many potential predisposing factors for PAONK, such as the use of irrigation liquid, a tourniquet, and various instruments, the preexisting or iatrogenic chondral damage, the aggressive postoperative rehabilitation, and so on, were previously pointed out [2]. However, the debate concerning the true predisposing factors for PAONK still continues. Recently, Oda et al. [11] reported that there was a significant correlation between the meniscal extrusion and the onset of SPONK. The meniscal extrusion is caused by root tear, radial tear, and degeneration of the meniscus [12–14]. It is known that the meniscal extrusion results in a loss of the normal meniscal functions and an increase of the contact load at the joint surface [15–19]. Therefore, there is a high probability that a significant relationship exists between the meniscal extrusion and the onset of PAONK. However, no studies have been conducted to analyze this relationship. Thus, the present study was conducted to analyze the relationship between the meniscal extrusion and the onset of PAONK. Out of 876 knees which had undergone arthroscopic partial meniscectomy of the medial meniscus (MM), 10 knees with PAONK were detected (PAONK group). Then, from the remaining 866 knees, in which PAONK did not occur after partial meniscectomy, the authors randomly selected 10 matched control knees. Namely, the gender, the age, and the body mass index of these 10 knees were matched with those of the PAONK group. The meniscus location on the joint line in the PAONK group was compared with that in the Control groups.

The purpose of the present study is to test the following hypotheses. (1) Before the arthroscopic partial meniscectomy, the MM might be slightly but significantly extruded in comparison with the LM in both the PAONK and Control groups. (2) Before the meniscectomy, the degree of the MM extrusion in the PAONK group might be significantly greater than that in the Control group. (3) After the meniscectomy, the degree of the MM extrusion might significantly increase in the PAONK group, while it might not significantly change in the Control group. Consequently, the degree of the MM extrusion might be significantly greater in the PAONK group than in the Control group.

## Methods

This series included 876 knees that underwent arthroscopic partial meniscectomy in Yagi Orthopaedic Hospital (Sapporo, Japan) between 2010 and 2015. This retrospective study design was accepted by the Ethical Review Board in this hospital. Out of the 876 knees, 10 knees with PAONK were detected within the 12-month follow-up period after the arthroscopic meniscectomy (PAONK group). PAONK was diagnosed with MRI examinations using the following criteria [2, 20]: (1) The patient complained of serious knee pain within 12 months after arthroscopic meniscectomy. (2) The MRI showed osteonecrosis in the femoral condyle or the tibial plateau at the ipsilateral side of the arthroscopic surgery. (3) It was confirmed that the MRI taken before the arthroscopic surgery did not show any evidence of BME or osteonecrosis. In the T1- and T2*-weighted MRI, PAONK showed the following findings: (1) In the early stages, a necrotic lesion was observed in the femoral condyle or tibial plateau as a low signal area surrounded by an extensive BME area. (2) At the margin of the necrotic lesion, a high signal line was often observed, delineating the necrotic area from the adjacent area of BME. (3) In the late stage, bone sequestration or segmentation was sometimes observed with a surrounding high signal rim in the flattened femoral condyle. Concerning these 10 knees (PAONK group), the clinical data were retrospectively collected from the medical records. Then, 10 matched control knees were randomly selected out of the remaining 866 knees without PAONK (Control group). Namely, the gender, the age, and the body mass index of these 10 knees were matched with those of the PAONK group. Then, their clinical data were collected from the medical records. The radiological stage of osteoarthritis (OA) and osteonecrosis was evaluated using the Kellgren and Lawrence (KL) grading system [21] and Koshino’s classification [22], respectively.

In both the PAONK and Control groups, each patient first underwent conservative treatments, based on the physical examinations and the radiograms. When the treatment was ineffective or when the knee pain recurred after temporary improvement, the MRI examination was carried out. The period from the onset of the knee pain to the MRI examination ranged from 2 to 32 weeks in the PAONK group (Table 1) and from 2 to 31 weeks in the Control group (Table 2). Various meniscal lesions were found by the MRI examinations in each group (Tables 3 and 4). Each patient hoped to undergo an arthroscopic surgery, after choices of the treatment for the meniscal lesions were informed. Consequently, arthroscopic meniscectomy was performed immediately (within 3 weeks) after the MRI examination.

**Table 1:** Demographic data before the meniscectomy for the 10 patients with PAONK. Summary of continuous variables is shown as “mean (standard deviation)”

Patient No.	Age Sex	Side	Body weight (Kg)	BMI (kg/m^2^)	Period from the onset of pain to the MRI ^a^	BME or osteonecrosis in MRI	Diagnosis (MRI, Arthroscopy)	OA grade	FTA
1.	70’s, F	L	70	30.3	12 wks	None	MMT, CM@MFC&MTP&PF	2	175^o^
2.	60’s, M	R	61	22.4	10 wks	None	MMT, CM@MFC&MTP	2	176^o^
3.	60’s, F	L	75	33.3	10 wks	None	MMT, CM@MFC&MTP	2	182^o^
4.	70’s, F	L	50	22.8	2 wks	None	MMT, CM@MFC&MTP	2	174^o^
5.	60’s, F	R	50	20.3	4 wks	None	MMT, CM@MFC&MTP&LTP&PF	2	178^o^
6.	60’s, F	L	52	21.6	14 wks	None	MMT, LMT, CM@MFC&PF	1	177^o^
7.	60’s, F	R	71	32.4	8 wks	None	MMT, CM@MFC&MTP	2	178^o^
8.	80’s, M	R	58	25.8	2 wks	None	MMT, CM@MFC&MTP	1	176^o^
9.	60’s, F	R	58	23.2	32 wks	None	MMT, LMT, CM@MFC&LTP&PF	2	168^o^
10.	60’s, F	R	64	26.6	31 wks	None	MMT, LMT, CM@MFC&MTP	0	178^o^
Summary	67.5		60.9	25.9	12.5 wks﻿		MMT: 10	G2: 7	176.2
	(5.9)		(8.5)	(4.4)	(10.2)		CM@MFC: 10	G1: 2	(3.4)
							CM@MTP: 8	G0: 1	

*F* Female, *M* Male, *R* Right, *L* Left, *BME* Bone marrow edema, *MMT* Medial meniscus tear, *LMT* Lateral meniscus tear, *CM* Chondromalacia, *MFC* Medial femoral condyle, *MTP* Medial tibial plateau, *LTP* Lateral tibial plateau, *PF* Patellofemoral, *OA* Osteoarthritis, *FTA* Femorotibial angle. ^a^ The period from the onset of the knee pain to the MRI examination

**Table 2:** Demographic data before the meniscectomy for the 10 matched control knees. Summary of continuous variables are shown as “mean (standard deviation)”

Patient No	Age Sex	Side	Body weight (Kg)	BMI (kg/m2)	Period from the onset of pain to the MRI^a^	BME or osteonecrosis in MRI	Diagnosis (MRI, Arthroscopy)	OA grade	FTA
1.	50’s, F	R	83	31.8	13 wks	None	MMT, LMT, CM@MFC&LTP &MTP	2	178^o^
2.	60’s, F	L	53	21.5	30 wks	None	MMT, CM@MFC&MTP&PF	2	176^o^
3.	60’s, F	L	57	24.8	26 wks	None	MMT, CM@MFC&MTP	1	175^o^
4.	60’s, F	R	70	28.0	31 wks	None	MMT, LMT, CM@MFC&LTP &MTP	1	173^o^
5.	60’s, F	L	66	28.9	8 wks	None	MMT, CM@MFC&MTP&PF	2	177^o^
6.	70’s, F	R	40	17.1	10 wks	None	MMT, LMT, CM@MFC&MTP&LTP&PF	3	181^o^
7.	70’s, F	R	48	20.0	3 wks	None	MMT, CM@MFC&MTP	2	178^o^
8.	70’s, F	L	54	23.7	16 wks	None	MMT, LMT, CM@MFC&LTP &MTP	2	174^o^
9.	80’s, M	L	60	21.6	2 wks	None	MMT, CM@MFC	2	177^o^
10.	60’s, M	L	88	31.2	4 wks	None	MMT, CM@MFC	1	174^o^
Summary	68		61.9	24.9	14.6 wks		MMT: 10	G3: 1	176.4
	(7.3)		(14.3)	(4.7)	(10.7)		CM@MFC: 10	G2: 6	(2.3)
							CM@MTP: 8	G1: 3	

*F* Female, *M* Male, *R* Right, *L* Left, *BME* Bone marrow edema, *MMT* Medial meniscus tear, *LMT* Lateral meniscus tear, *CM* Chondromalacia, *MFC* Medial femoral condyle, *MTP* Medial tibial plateau, *LTP* Lateral tibial plateau, *PF* Patellofemoral, *OA* Osteoarthritis, *FTA* Femorotibial angle. ^a^ The period from the onset of the knee pain to the MRI examination

**Table 3:** Intra- and post-operative data for the 10 patients with PAONK. Summary of continuous variables is shown as “mean (standard deviation)”

Patient No	Age Sex	Type of MM tear	Surgery Done	Resected width of MM	Postop. period until the pain recurrence ^a^	Postop. period until taking the MRI^b^
1.	70’s, F	RT at PH (with HT)	PMM	Subtotal	8 wks	12 wks
2.	60’s, M	RT at PH	PMM	Half	12 wks	19 wks
3.	60’s, F	PRT	PMM	Subtotal	2 wks	4 wks
4.	70’s, F	PRT (with HT)	PMM	Subtotal	4 wks	6 wks
5.	60’s, F	PRT (with HT)	PMM	Subtotal	8 wks	9 wks
6.	60’s, F	PRT	PMM, PLM	Subtotal	6 wks	11 wks
7.	60’s, F	RT at PH	PMM	Half	7 wks	21 wks
8.	80’s, M	RT at PH (with HT)	PMM	Subtotal	7 wks	19 wks
9.	60’s, F	PRT	PMM, PLM	Subtotal	3 wks	5 wks
10.	60’s, F	RT at PH (with HT)	PMM, LMR	Subtotal	Unclear	3 wks
Summary		PRT: 5	PMM: 10	Subtotal: 8	5.7 wks﻿	12.1 wks﻿
		RT: 5	PLM: 2 LMR: 1	Half: 2	(3.3)	(7.9)

*F* Female, *M* Male, *MM* Medial meniscus, *RT* Radial tear, *PH* Posterior horn, *HT* Horizontal tear, *PRT* Posterior root tear, *PMM* Partial medial meniscectomy, *PLM* Partial lateral meniscectomy, *LMR* Lateral meniscus repair. ^a^ The period from the meniscectomy to the recurrence of knee pain. ^b^ The period from the meniscectomy to the time of diagnosis of PAONK using MRI

**Table 4:** Intra- and post-operative data for the 10 matched control knees. Summary of continuous variables is shown as “mean (standard deviation)”

Patient No	Age Sex	Type of MM tear	Surgery Done	Resected width of MM	Postoperative period until taking the MRI^a^
1.	50’s, F	HT at Posterior	PMM, PLM	Half	11 wks
2.	60’s, F	HT at Posterior	PMM	Half	6 wks
3.	60’s, F	HT at Posterior	PMM	Half	17 wks
4.	60’s, F	RT with HT at Posterior	PMM, PLM	Half	4 wks
5.	60’s, F	HT at Posterior	PMM, PLM	Half	3 wks
6.	70’s, F	HT at Posterior	PMM	Half	13 wks
7.	70’s, F	RT with HT at Posterior	PMM	Half	35 wks
8.	70’s, F	RT with HT at Posterior	PMM, PLM	Half	50 wks
9.	80’s, M	RT at Posterior	PMM	Half	12 wks
10.	60’s, M	RT with HT at Posterior	PMM	Half	13 wks
Summary		HT: 5	PMM: 10	Subtotal: 0	16.6 wks
		RT: 5	PLM: 4	Half: 10	(14.3)

*F* Female, *M* Male, *MM* Medial meniscus, *RT* Radial tear, *PH* Posterior horn, *HT* Horizontal tear, *PRT* Posterior root tear, *PMM* Partial medial meniscectomy, *PLM* Partial lateral meniscectomy, *LMR* Lateral meniscus repair. ^a^ The period between the meniscectomy and the time of taking the postoperative MRI

In the PAONK group, the postoperative MRI was taken at the time range from 3 to 21 weeks (mean, 12.1 weeks; standard deviation, 7.9) after the meniscectomy (Table 3). The diagnosis of PAONK was made with this MRI. In the Control group, the postoperative MRI was taken at the time range from 3 to 50 weeks (mean, 16.6 weeks; standard deviation, 14.3) after the meniscectomy (Table 4). In this group, the postoperative MRI was taken to diagnose causes of the various symptoms in the knees, which were experienced by the patients. There was no significant difference between the 2 groups concerning the period between the meniscectomy and the postoperative MRI.

**Fig. 1:**
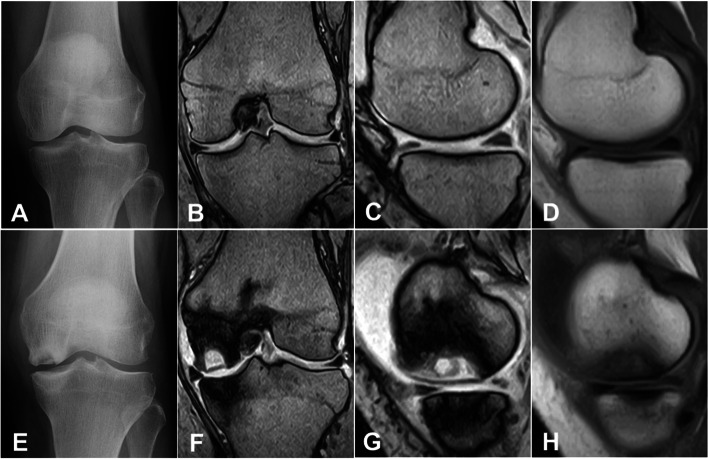
62-year old woman. Preoperatively, the radiogram (A) showed grade-1 OA. The images taken with the 0.3-T MRI (B and C: T2*, D: T1) did not show any findings of osteonecrosis or bone marrow edema (BME). At 3 months after the meniscectomy, the radiograms (E) indicated stage-3 osteonecrosis in the medial femoral condyle. MR images (F and G: T2*, H: T1) showed a necrotic lesion, which was surrounded by an osteosclerotic zone and a wide BME area. The resolution of the MR images was acceptable to measure the meniscus location on the joint line

In this study, 0.3-T MRI (Aris; Hitachi, Tokyo, Japan) was used. For T1-weighted images, the protocol included both sagittal and coronal spin-echo sections with TR/TE values of 550 per 27 milliseconds. For T2*-weighted images, gradient-echo sections with TR/TE of 660 per 17 milliseconds (FA of 30 degrees) were used. Section thickness was between 4 and 5 mm with 4-mm intervals. An extremity coil was used with a field of view of 150 mm, 256 × 256 matrix. Representative MR images used in this study were shown in Fig. 1. The resolution of the images was acceptable not only to detect PAONK but also to measure the meniscus location on the joint line (Fig. 1).

**Fig. 2:**
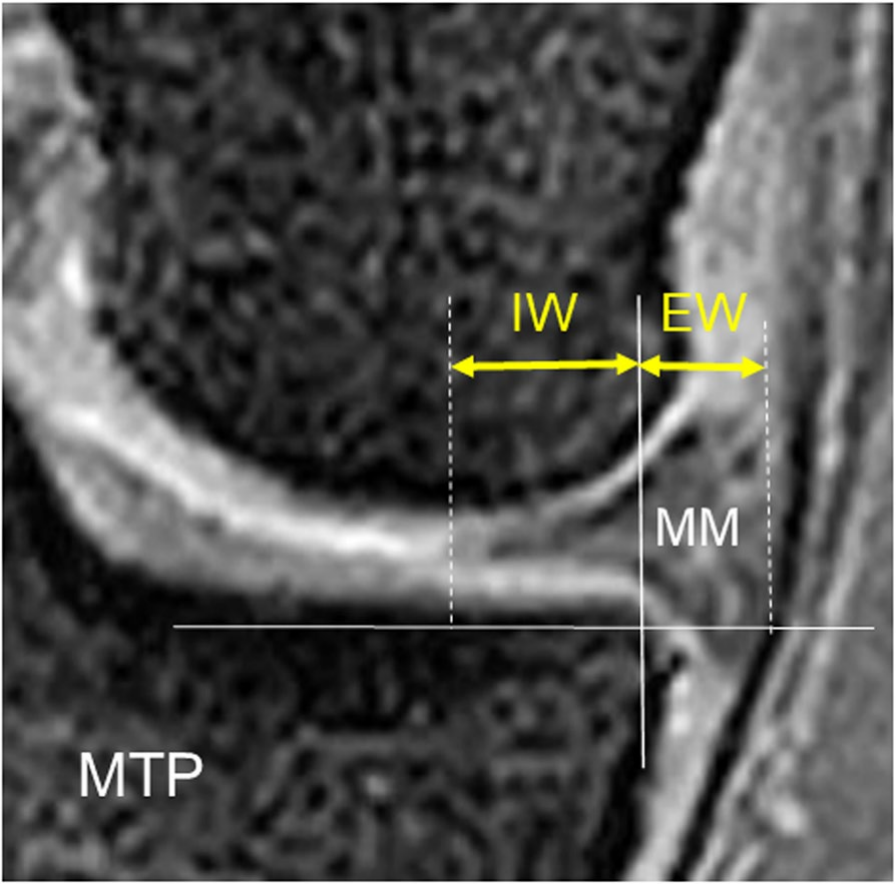
Measurement of the location of the medial meniscus (MM) on the coronal image of the MRI. A vertical line intersecting the peripheral margin of the medial tibial plateau (MTP) was drawn. Then, the second and third lines, which were parallel to the first line, were drawn at the outer and inner margins of the meniscus, respectively. The distance between the first and second lines, which was defined as “Extrusion width (EW)”, and the distance between the first and third lines, which was defined as “Inner width (IW)”, were measured. Each width was quantified in millimeters

The location of the MM on the joint line was quantified using the modified Costa’s method [12]. On the coronal image of the MRI, which was sectioned at the midpoint of the medial tibial plateau, a researcher drew a vertical line intersecting the peripheral margin of the medial tibial plateau (Fig. 2). Then, the second and third lines, which were parallel to the first line, were drawn at the outer (peri-articular) and inner (intra-articular) margins of the meniscus, respectively (Fig. 2). The researcher measured the distance between the first and second lines, which was defined as “Extrusion width (EW)”, and the distance between the first and third lines, which was defined as “Inner width (IW)” (Fig. 2). This method was applied to the lateral joint space in the same manner to quantify the location of the LM. In each group, the location of the MM and the LM on the joint line was independently measured by 3 orthopaedic surgeons (JY, TK, and KY). The intra- and inter-rater reliability was evaluated by calculating the intra- and inter-class coefficients, based on confirmation of the normality using the Shapiro-Wilk test.

To statistically compare each parameter between the PAONK and Control groups, the Wilcoxon matched-pairs signed-ranks test, the Mann-Whitney U test, Fisher’s exact test, and chi-square test for trend, which were non-parametric tests, were used. The calculation was made with IBM SPSS Statistics, version-23 (IBM Corp, Armonk, New York). The significance level was set at *P* < 0.05.

## Results

Concerning the PAONK group, the demographic data before the meniscectomy are shown in Table 1. The preoperative femorotibial angle was 176.2 ± 3.4 degrees with a range from 168 to 182 degrees (Table 1). The intra- and post-operative data are shown in Table 3. In the 10 knees, 5 knees had a posterior root tear of the MM, and the other 5 knees had a radial tear at the posterior horn of the MM (Table 3). In these knees, meniscus degeneration was widely found around the torn portion. Therefore, the posterior horn around the torn portion was almost totally resected in 8 knees during the arthroscopic meniscectomy, while a half width of the posterior horn was resected in the remaining 2 knees (Table 3). Characteristics of the osteonecrosis lesions in the PAONK group are shown in Table 5. An osteonecrosis lesion was found at the medial femoral condyle (MFC) in 8 knees and at both the MFC and the medial tibial plateau (MTP) in 2 knees (Table 5). The osteonecrosis lesions were classified as Stage 1 in 3 knees, Stage 2 in 5 knees, and Stage 3 in 2 knees (Table 5).

**Table 5:** Characteristics of the osteonecrosis lesions in the 10 knees with PAONK. Summary of continuous variables is shown as “mean (standard deviation)”

Patient No.	Age Sex	Location of the necrotic lesion	Area of BME	Necrosis stage (Koshino)	OA grade (KL)	FTA	Subsequent Treatment
1.	70’s, F	MFC	Narrow	S-2	G-2	176	TKA
2.	60’s, M	MFC	Moderate	S-2	G-3	178	HTO, OATS
3.	60’s, F	MFC	Wide	S-2	G-2	181	UKA
4.	70’s, F	MFC	Narrow	S-1	G-3	175	UKA
5.	60’s, F	MFC	Narrow	S-2	G-2	179	UKA
6.	60’s, F	MFC	Moderate	S-3	G-1	177	UKA
7.	60’s, F	MFC	Wide	S-3	G-3	177	UKA
8.	80’s, M	MFC&MTP	Narrow	S-2	G-3	178	TKA
9.	60’s, F	MFC	Narrow	S-1	G-2	169	TKA
10.	60’s, F	MFC&MTP	Moderate	S-1	G-0	178	UKA
Summary		MFC: 8	Narrow: 5	S-1: 3	G-0: 1	177	UKA: 6
		MFC&MTP: 2	Moderate: 3	S-2: 5	G-1: 1	(3.0)	TKA: 3
			Wide: 2	S-3: 2	G-2: 4		HTO: 1
					G-3: 4		

*F* Female, *M* Male, *MFC* Medial femoral chondyle, *MTP* Medial tibial plateau, *BME* Bone marrow edema, *OA* Osteoarthritis, *UKA* Unicompartmental knee arthroplasty, *TKA* Total knee arthroplasty, *OATS* Osteochondral autograft transfer system, *HTO* High tibial osteotomy

Regarding the Control group, the demographic data before the meniscectomy are shown in Table 2. Five knees had a radial tear at the posterior horn of the MM, and the other 5 knees had a horizontal tear at the posterior horn of the MM (Table 4). It was noted that there was no posterior root tear of the MM. The intra- and post-operative data are shown in Table 4. In these knees, approximately a half width of the posterior horn was resected in the meniscectomy (Table 4). Comparisons of these data between the PAONK and Control groups are summarized in Table 6. There were no significant differences in all factors between the 2 groups except for two, the type of the MM tear (*P* = 0.0016) and the resected width of the MM (*P* = 0.0007) (Table 6).

**Table 6:** Comparisons between the PAONK and Control groups concerning the preoperative, surgical, and post-operative factors. Continuous variables are shown as “mean (standard deviation)”

Factors	PAONK group	Control group	Comparison
Age	67.5 (5.9)	68.0 (7.3)	N.S.*
Sex	M: 2, F: 8	M: 2, F: 8	N.S.**
Body weight	60.9 (8.5)	61.9 (14.3)	N.S.*
BMI (kg/m^2^)	25.9 (4.4)	24.9 (4.7)	N.S.*
OA grade	G0: 1, G1: 2, G2: 7	G1: 3, G2: 6, G3: 1	N.S.***
FTA	176.2 (3.4)	176.4 (2.3)	N.S.*
Period for preoperative conservative treatment	12.5 wks (10.2)	14.6 wks (10.7)	N.S.*
Diagnosis	MMT: 10, CM@MFC: 10	MMT: 10, CM@MFC: 10	N.S.***
	CM@MTP: 8	CM@MTP: 8	
Type of MM tear	PRT: 5, RT: 5	RT: 5, HT: 5	*P* = 0.0016**
Surgery done	PMM: 10, PLM: 2, LMR: 1	PMM: 10, PLM: 4	N.S.***
Resected width of MM	Subtotal: 8, Half: 2	Subtotal: 0, Half: 10	*P* = 0.0007**
Period from the surgery to the postoperative MRI	12.1 wks (7.9)	16.6 wks (14.3)	N.S.*

*F* Female, *M* Male, *R* Right, *L* Left, *BME* Bone marrow edema, *MMT* Medial meniscus tear, *LMT* Lateral meniscus tear, *CM* Chondromalacia, *MFC* Medial femoral condyle, *MTP* Medial tibial plateau, *LTP* Lateral tibial plateau, *OA* osteoarthritis, *FTA* Femorotibial angle. * Mann-Whitney U test. ** Fisher’s exact test. *** Chi-square test for trend

Concerning the location of the MM on the joint line before and after the meniscectomy, the EW and IW values in the PAONK and Control groups were shown in Tables 7 and 8, respectively, and the summarized data for comparisons between the 2 groups were shown in Table 9. Before the meniscectomy, the EW of the MM in the PAONK group (4.7 ± 1.4 mm) was significantly greater (*P* = 0.0195) than that in the Control group (3.0 ± 1.3 mm) (Table 9). In each group, the preoperative EW value was significantly greater (*P* < 0.0001 for the PAONK group and *P* = 0.0002 for the Control group) than that of the LM values (− 0.2 ± 0.8 and 0.1 ± 1.2 mm, respectively) (Tables 7 and 8). After the meniscectomy, the EW of the MM in the PAONK group (5.9 ± 1.1 mm) was significantly greater (*P* = 0.0009) than that in the Control group (3.4 ± 1.4 mm) (Table 9).

**Table 7:** The Extrusion width (EW) and the Inner width (IW) of the medial meniscus (MM) and the lateral meniscus (LM), which were measured before meniscectomy and at the time when PAONK was diagnosed. In the “Changes between the 2 periods”, plus and minus values show an increase and a decrease of the width, respectively

Patient No.	Age Sex	Before meniscectomy				At the time of diagnosis of PAONK				Changes between the 2 periods			
		EW (mm)		IW (mm)		EW (mm)		IW (mm)		EW (mm)		IW (mm)	
		MM	LM	MM	LM	MM	LM	MM	LM	MM	LM	MM	LM
1.	70’s, F	7.1	0.0	2.0	8.0	8.0	0.0	−1.6	7.9	0.9	0.0	3.6	- 0.1
2.	60’s, M	4.1	−0.6	6.9	10.8	4.2	−0.6	1.2	11.0	0.1	0.0	5.7	0.2
3.	60’s, F	4.4	0.4	4.9	8.1	5.1	0.4	3.5	8.1	0.7	0.0	1.4	0.0
4.	70’s, F	6.8	−0.4	2.9	8.6	6.8	− 0.4	0.0	8.5	0.0	0.0	2.9	−0.1
5.	60’s, F	5.4	−0.6	0.0	8.1	6.6	−0.6	−1.2	8.5	1.2	0.0	1.2	0.4
6.	60’s, F	3.5	1.3	6.1	7.2	6.1	1.3	1.4	7.0	2.6	0.0	4.7	−﻿0.2
7.	60’s, F	5.8	0.0	1.7	7.2	6.1	0.0	−2.1	7.8	0.3	0.0	3.8	0.6
8.	80’s, M	3.7	−1.1	6.5	12.7	5.9	−1.3	0.6	13.3	2.2	−0.2	5.9	0.6
9.	60’s, F	2.8	−1.8	6.1	10.6	4.8	−1.8	1.6	7.9	2.0	0.0	4.5	−2.7
10.	60’s, F	3.4	0.6	7.1	9.4	4.9	0.8	2.2	9.0	1.5	0.2	4.9	−0.4
Mean		4.7	−0.2	4.4	9.1	5.9	−0.2	0.6	8.9	1.2	0.0	3.9	−0.2
(SD)		(1.4)	(0.8)	(2.4)	(1.7)	(1.1)	(0.9)	(1.7)	(1.8)	(0.9)	(0.1)	(1.5)	(0.9)
*P* value		*P* < 0.0001^a^		*P* < 0.0001 ^a^		*P* < 0.0001 ^a^		*P* < 0.0001 ^a^		*P* = 0.0039 ^b^	N.S. ^b^	*P* = 0.0020 ^b^	N.S. ^b^

^a^ comparison between the MM and the LM (Mann-Whitney U test). ^b^ comparison between the 2 periods (Wilcoxon matched-pairs signed rank test)

**Table 8:** The Extrusion width (EW) and the Inner width (IW) of the medial meniscus (MM) and the lateral meniscus (LM) in the 10 matched control knees. The measurements were performed on the MR images taken before meniscectomy and at the time in a range from 3 to 50 weeks after the meniscectomy. In the “Changes between the 2 periods”, plus and minus values show an increase and a decrease of the width, respectively

Patient No.	Age Sex	Before meniscectomy				1 year after meniscectomy				Changes between the 2 periods			
		EW (mm)		IW (mm)		EW (mm)		IW (mm)		EW (mm)		IW (mm)	
		MM	LM	MM	LM	MM	LM	MM	LM	MM	LM	MM	LM
1.	50’s, F	1.4	1.6	7.8	10.5	1.6	1.8	5.7	9.2	0.2	0.2	2.1	1.3
2.	60’s, F	2.8	−1.2	5.6	7.9	2.8	−1.6	3.6	7.8	0.0	−0.4	2.0	0.1
3.	60’s, F	1.4	−0.3	8.7	12.5	1.5	0.0	4.7	12.4	0.1	0.3	4.0	0.1
4.	60’s, F	4.4	1.9	5.2	5.2	4.6	2.2	2.4	5.2	0.2	0.3	2.8	0.0
5.	60’s, F	1.8	0.2	6.7	10.1	2.2	0.0	3.8	9.8	0.4	−0.2	2.9	0.3
6.	70’s, F	3.4	0.8	4.1	9.1	3.5	1.0	3.2	8.9	0.1	0.2	0.9	0.2
7.	70’s, F	2.7	−2.2	3.9	12.5	2.8	−2.1	2.7	12.6	0.1	0.1	1.2	−0.1
8.	70’s, F	2.9	0.6	8.5	9.7	3.5	1.1	4.8	7.1	0.6	0.5	3.7	2.6
9.	80’s, M	3.9	−0.6	7.2	15.3	5.5	−0.4	4.4	15.6	1.6	0.2	2.8	−0.3
10.	60’s, M	5.5	0.0	5.2	15.0	5.6	−0.1	3.3	15.3	0.1	−0.1	1.9	−0.3
Mean		3.0	0.1	6.3	10.8	3.4	0.2	3.9	10.4	0.3	0.1	2.4	0.4
(SD)		(1.3)	(1.2)	(1.7)	(3.0)	(1.4)	(1.3)	(1.0)	(3.3)	(0.5)	(0.3)	(0.9)	(0.9)
*P* value		*P* = 0.0002 ^a^		*P* = 0.0010 ^a^		*P* = 0.0002 ^a^		*P* < 0.0001 ^a^		*P* = 0.0039 ^b^	N.S. ^b^	*P* = 0.0020 ^b^	N.S. ^b^

^a^ comparison between the MM and the LM (Mann-Whitney U test). ^b^ comparison between the 2 periods (Wilcoxon matched-pairs signed rank test)

**Table 9:** The Extrusion width (EW), Inner width (IW), and Total width (EW + IW) of the medial and lateral menisci were compared between the PAONK group and the matched control group at the 2 periods, before meniscectomy and at the time of diagnosis of PAONK, respectively. The comparisons were made using Mann-Whitney U test. Concerning the time of the MRI examination, “After meniscectomy^a^” means “At the time of diagnosis of PAONK” in the PAONK group and “At the time of the second-look MRI taken 6 – 12 months after the meniscectomy” in the Control group

Meniscus	Measures	Time of the MRI examination	PAONK Group	Control Group	Comparison
**Medial**	Extrusion width (EW)	Before meniscectomy	4.7 (1.4)	3.0 (1.3)	*P* = 0.0195
		After meniscectomy^a^	5.9 (1.1)	3.4 (1.4)	*P* = 0.0009
	Inner width (IW)	Before meniscectomy	4.4 (2.4)	6.3 (1.7)	N.S.
		After meniscectomy^a^	0.6 (1.7)	3.9 (1.0)	*P* = 0.0001
	Total width (EW + IW)	Before meniscectomy	9.1 (1.5)	9.3 (1.5)	N.S.
		After meniscectomy^a^	6.4 (1.2)	7.2 (1.3)	N.S.
**Lateral**	Extrusion width (EW)	Before meniscectomy	−0.2 (0.8)	0.1 (1.2)	N.S.
		After meniscectomy^a^	−0.2 (0.9)	0.2 (1.3)	N.S.
	Inner width (IW)	Before meniscectomy	9.1 (1.7)	10.8 (3.0)	N.S.
		After meniscectomy^a^	8.9 (1.8)	10.4 (3.3)	N.S.
	Total width (EW + IW)	Before meniscectomy	8.9 (1.3)	10.9 (2.6)	N.S.
		After meniscectomy^a^	8.7 (1.6)	10.6 (2.9)	N.S.

Mean (standard deviation)

The mean IW of the MM in the PAONK group was 4.4 ± 2.4 mm before the meniscectomy, and it significantly decreased (*P* = 0.0020) to 0.6 ± 1.7 mm after the meniscectomy (Table 7). In the Control group, the mean IW of the MM significantly decreased (*P* = 0.0020) from 6.3 ± 1.7 to 3.9 ± 1.0 mm after the meniscectomy (Table 8). The IW value after the meniscectomy was significantly less (*P* = 0.0001) in the PAONK group than that in the Control group (Table 9). Regarding the total width of the MM after the meniscectomy, there were no significant differences between the PAONK and Control groups (6.4 ± 1.2 and 7.2 ± 1.3 mm, respectively) (Table 9).

Concerning reliability of the measurement, the intra-class coefficients on the EW and the IW were 0.950 (95% confidence level 0.896–0.979) and 0.994 (0.987–0.997), respectively. The inter-class coefficients on the EW and the IW were 0.865 (0.74–0.939) and 0.98 (0.96–0.99), respectively. Thus, the intra- and inter-rater reliabilities for the measurement of the EW and the IW were found to be satisfactory.

## Discussion

The most important findings of the present study were as follows: (1) Before the arthroscopic partial meniscectomy, the MM was slightly but significantly extruded in comparison with the LM, which was normally located, in both the PAONK and Control groups. (2) Before the meniscectomy, the degree of the MM extrusion in the PAONK group was significantly greater than that in the Control group. (3) After the meniscectomy, the degree of the MM extrusion significantly increased in the PAONK group so that the IW of the MM became almost lost, while the degree of the MM extrusion did not significantly change in the Control group. (4) Consequently, the degree of the MM extrusion was significantly greater in the PAONK group than in the Control group. The authors considered why the meniscus extrusion increased after meniscectomy, particularly in the PAONK group. Before the meniscectomy, the EW and IW values was 4.7 ± 1.4 and 4.4 ± 2.4 mm in the PAONK group. This fact shows that the circumferential fibers in the peripheral portion of the MM were damaged but not completely torn before the meniscectomy. In such a pre-condition of the MM, subtotal resection was performed in 8 knees. Therefore, the authors considered that, in the PAONK group, the residual circumferential fibers in the peripheral portion of MM might be completely resected by the meniscectomy, resulting in the increase of the mean EW from 4.7 ± 1,4 to 5.9 ± 1,1 mm. On the other hand, all knees underwent a half width resection in the Control knee. The authors considered that, in the Control group, a majority of the residual circumferential fibers located in the peripheral portion of the MM might remain preserved, resulting in the limited increase of the EW values from 3.0 ± 1.3 to 3.4 ± 1.4 mm.

**Fig. 3:**
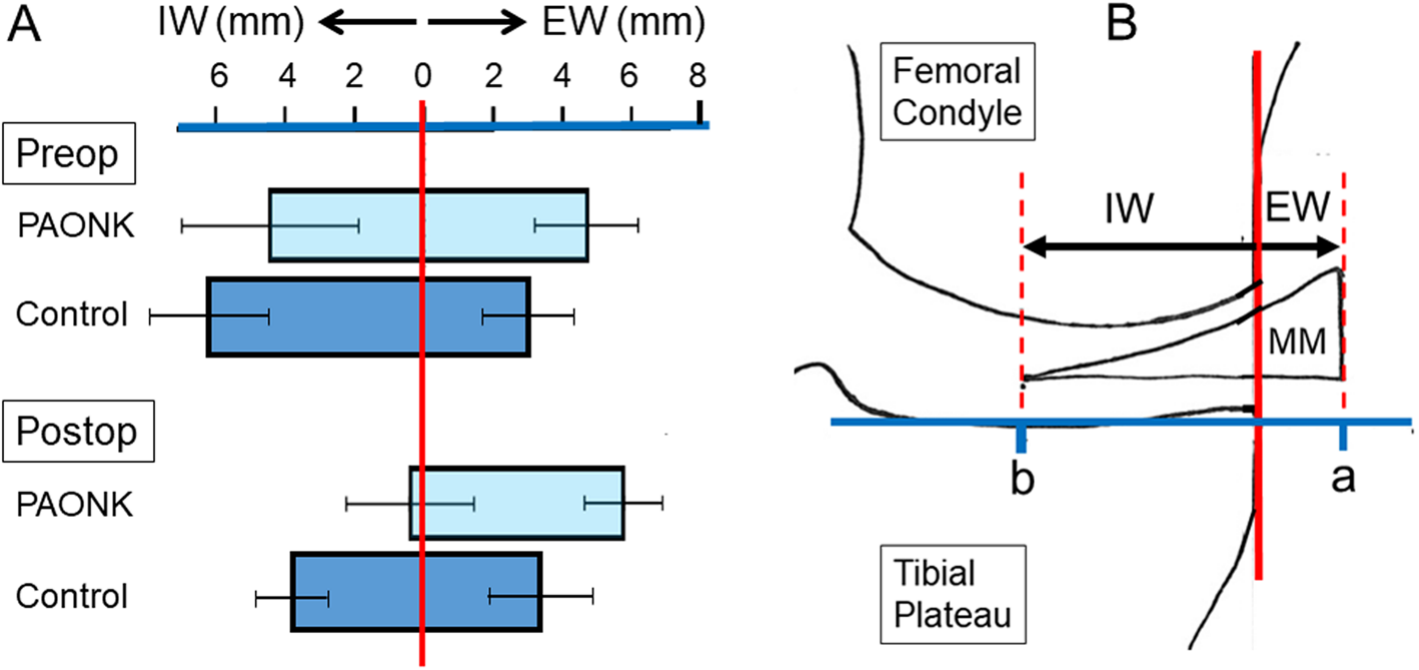
The left graph (**A**) shows location of the MM on the joint line in the PAONK and Control groups, which were measured before and the meniscectomy (Preop and Postop). The right schematic picture (**B**) shows the definition of the Extrusion width (EW) and the Inner width (IW) and how to understand the left graph. This left graph shows that the disappearance of the IW of the MM in the PAONK group was caused by not only the decrease of the total meniscal width due to meniscectomy but also the meniscal extrusion

In addition, the present study showed that the IW of the MM in the PAONK group was significantly less than that in the Control group after the meniscectomy. Namely, the IW of the MM was almost lost (mean 0.6 ± 1.7 mm) in the PAONK group after the meniscectomy, while it averaged 3.9 mm in the Control group. On the other hand, no significant difference was detected between the 2 groups concerning the total width of the MM measured after the meniscectomy, although the total width was reduced by approximately 2 mm due to the meniscectomy in both groups. Figure 3 shows a schematic picture of the phenomena that occurred in the MM due to the meniscectomy performed in the 2 groups. Figure 3 explains that the significant difference in the IW of the MM was mainly caused by the difference in the degree of the MM extrusion.

The above-described results in the present study showed that there was a significant relationship between the degree of the MM extrusion and the onset of PAONK. Recently, a few studies suggested that the meniscus extrusion is a predisposing factor for SPONK [19, 23]. No studies, however, have studies the relationship between the meniscus extrusion and the onset of PAONK. This study first suggested that the extrusion of the MM is a potential predisposing factor for post-arthroscopy osteonecrosis of the knee.

The authors considered the mechanism of the onset of PAONK due to the extrusion of the MM. The present study showed that, in the knees with PAONK, the MM was significantly extruded so that the IW of the MM was almost complete lost. It is known that contact load at the medial joint surface excessively increases, particularly in varus knees [15–19]. Recently, a few studies have pointed out that an essential pathology of PAONK is a subchondral bone fracture, and that the necrotic bone lesion is a secondary condition of the fractured bone [10, 24, 25]. Therefore, the authors considered that the excessive loading at the joint surface might induce an insufficiency fracture of the subchondral bone, progressing to PAONK [26, 27]. This consideration is supported by the following fact observed in the present study. Namely, the BME lesion, which commonly shows the existence of an insufficiency fracture, was widely observed on the MRIs taken after the meniscectomy in each knee with PAONK.

There are limitations in the present study. First, because PAONK is a rare condition, the number of patients with PAONK was very limited so that the statistical power was insufficient. However, statistical differences were detected concerning the MM extrusion in comparison with the matched Control group. Secondly, the strength of magnetic field of the used MRI was 0.3 Tesla, and the section thickness was between 4 and 5 mm with 4-mm intervals. However, the resolution of the images was high enough to measure the meniscus location on the joint line, as seen in Fig. 1, so that the intra- and inter-rater reliabilities were satisfactory as shown in the result section. Thirdly, this was a retrospective study. However, the authors believe that this study is of value because this study showed the necessity to conduct a prospective comparative study using a greater number of subjects in the near future to confirm the role of the meniscus extrusion in the onset of PAONK.

As for clinical relevance, first, orthopaedic surgeons should be aware that arthroscopic meniscectomy for elder patients has a high risk of failure due to various causes including PAONK [28]. In the clinical field, however, there are many elder patients who need surgical treatments for their meniscus lesions. For such cases, meniscal repair should be performed, if it is possible, rather than the meniscectomy to avoid the postoperative meniscal extrusion [29]. However, it is also a fact that there are many knees in which arthroscopic partial meniscectomy of the MM is unavoidable. For such knees with partial meniscectomy of the MM, this study suggested that orthopaedic surgeons should carefully follow them up after the meniscectomy to diagnose the potential onset of PAONK as early as possible, particularly in the knees with MM extrusion.

## Conclusion

Before the meniscectomy, the Extrusion width of the MM was significantly greater than that in the Control group. In the MRI taken in a range from 1 to 50 weeks after the meniscectomy, the Extrusion width of the MM in the PAONK group was significantly greater than that in the Control group, and the Inner width of the MM in the PAONK group was significantly less than that in the Control group. This study suggests that the post-operative progression of the MM extrusion, which was induced by the arthroscopic meniscectomy, is a potential predisposing factor for PAONK.

## Data Availability

The datasets generated and/or analyzed during the current study are available from the corresponding author on reasonable request.

## Abbreviations

BME: Bone marrow edema
FTA: Femorotibial angle
KL: Kellgren and Lawrence
LM: Lateral meniscus
MFC: Medial femoral condyle
MM: Medial meniscus
MRI: Magnetic resonance imaging
MTP: Medial tibial plateau
OA: Osteoarthritis
PAONK: Post-arthroscopic osteonecrosis of the knee
SPONK: Spontaneous osteonecrosis of the knee
